# Internuclear diffusion of histone H1 within cellular compartments of *Aspergillus nidulans*

**DOI:** 10.1371/journal.pone.0201828

**Published:** 2018-08-16

**Authors:** Alexander P. Mela, Michelle Momany

**Affiliations:** Fungal Biology Group and Department of Plant Biology, University of Georgia, Athens, Georgia, United States of America; University of Minnesota, UNITED STATES

## Abstract

Histone H1 is an evolutionarily conserved linker histone protein that functions in arranging and stabilizing chromatin structure and is frequently fused to a fluorescent protein to track nuclei in live cells. In time-lapse analyses, we observed stochastic exchange of photoactivated Dendra2-histone H1 protein between nuclei within the same cellular compartment. We also observed exchange of histones between genetically distinct nuclei in a heterokaryon derived from fusion of strains carrying histone H1-RFP or H1-GFP. Subsequent analysis of the resulting uninucleate conidia containing both RFP- and GFP-labeled histone H1 proteins showed only parental genotypes, ruling out genetic recombination and diploidization. These data together suggest that the linker histone H1 protein can diffuse between non-daughter nuclei in the filamentous fungus *Aspergillus nidulans*.

## Introduction

Histones are members of an ancient family of proteins whose roles have been constantly redefined since their discovery. These proteins were originally thought to function solely in providing structural support for DNA organization and packaging within the nucleus, but recent work has revealed a broad range of intracellular and extracellular functions, including involvement in innate immune response, apoptosis induction, and cellular signaling [1–3]. The histones are classified as core (H2A, H2B, H3, and H4) or linker (H1 and H5) proteins, with linker histones showing greater movement within the nucleus than core histones. The dynamics of core histone transport have been characterized primarily in animal models, but the dynamics of linker histones are not as well-understood [1].

A series of classic pulse-chase experiments showed transport of radio-labeled linker histones H1 and H5 from human nuclei into inactive avian nuclei within fused HeLa cells. These experiments demonstrated that internuclear linker histone migration can occur independently of de novo synthesis in an artificial system [1,4]. Here we investigate internuclear histone H1 transport in the filamentous fungus *Aspergillus nidulans*. In *A*. *nidulans* multiple nuclei undergo ‘semi-open’ mitosis (partial disassembly of the nuclear pore complex) within a single cellular compartment [5]. *A*. *nidulans* can also undergo anastomosis (cell fusion) and plasmogamy (cytoplasmic fusion) without karyogamy (nuclear fusion), to form heterokaryons, in which genetically distinct nuclei co-exist in the same cytoplasm. Recently a study highlighting the movement of nuclei between newly forming colonies in *Fusarium* reported occasional colocalization of histone H1 proteins within heterokaryons, though this study was not designed to look at histone movement specifically [6]. Interestingly, Roper et al., conducted similar heterokaryon experiments in the filamentous fungus *N*. *crassa* and speculated that histone H1 might be diffusing between nuclei. The authors concluded that histone H1 diffusion among nuclei did not explain the mixing of nucleotypes in fungal chimeras, and that cytoplasmic flow was the major homogenizing force in heterokaryotic hyphae of *N*. *crassa* [7]. In *N*. *crassa*, many more nuclei populate hyphal compartments and nuclear division is closed and asynchronous, as opposed to the semi-open and parasynchronous nuclear division in *A*. *nidulans* [8–10]. To the best of our knowledge, our current manuscript is the first investigation specifically designed to study the movement of histone H1 between nuclei in a filamentous fungi which undergoes this type of mitosis and nuclear movement. Here we exploit the unique biology of *A*. *nidulans* to show that internuclear transport of histone H1 occurs naturally between non-daughter nuclei in this filamentous fungus.

## Results and discussion

**Fig 1:**
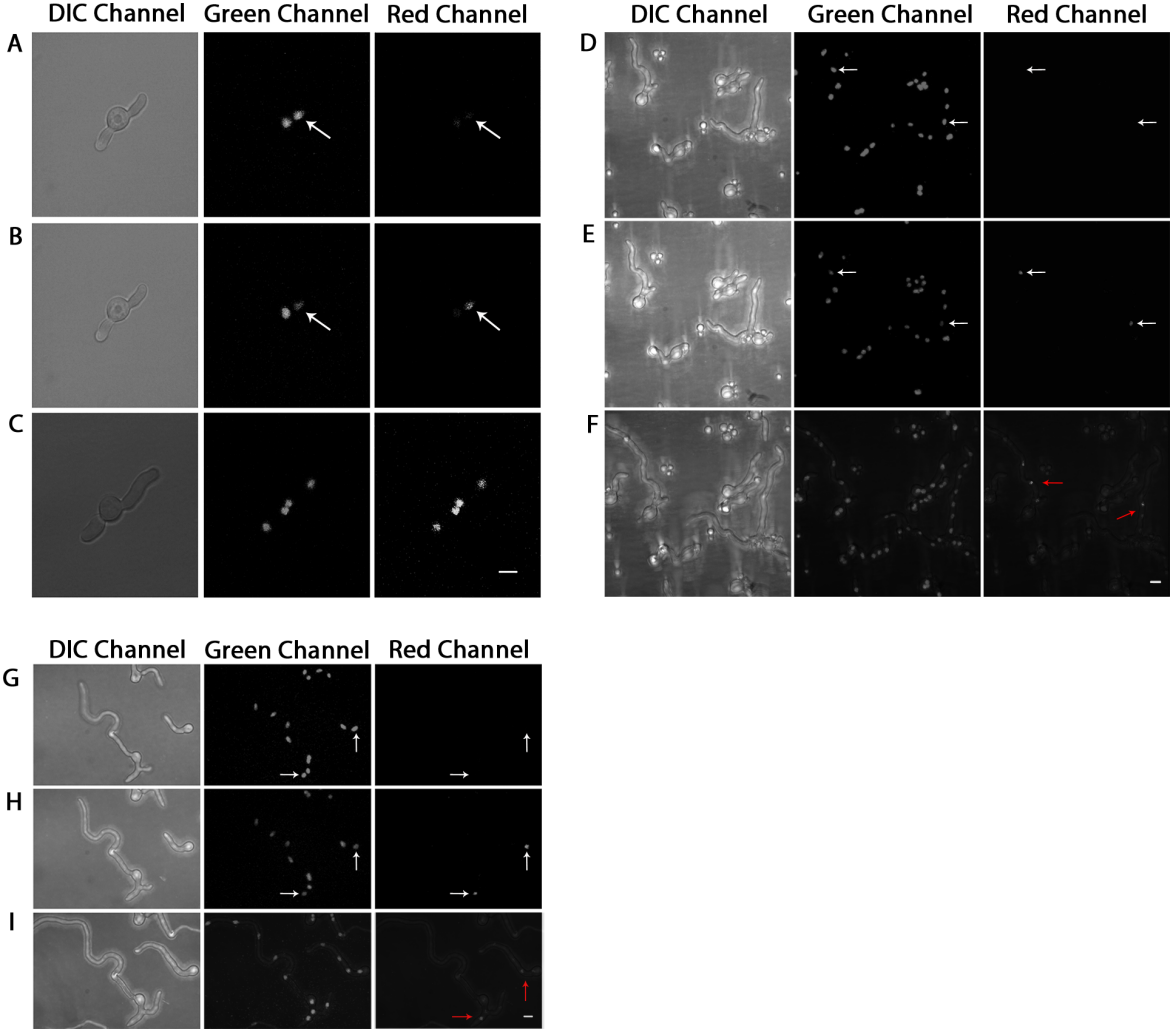
Histone H1 diffusion between nuclei can occur within cellular compartments. Dendra-H1-tagged nuclei in three independent experiments, shown in DIC, green, and red channels respectively, were photoconverted using 405nm laser on an LSM-510 confocal fluorescence microscope under 100X objective lens. (A,D,G) Shows pre-photoconversion; (B,E,H) post-photoconversion; (C) post-photoconversion after approximately 3 hours have elapsed; (F) post-photoconversion after approximately 3 hours have elapsed; (I) post-photoconversion after approximately 2.75 hours have elapsed. White arrows denote nuclei which have been selected for photoactivation. Red arrows denote the location of nuclei post photoactivation. Brightness increased for entire panel images accordingly to improve visualization. White Scale Bars = 5μm.

When a dormant asexual spore (conidium) of *A*. *nidulans* is furnished with a carbon source, it breaks dormancy, begins to swell, and a germ tube emerges. The single nucleus within the conidium undergoes multiple rounds of mitosis before a septum is formed, dividing the growing fungus into two asymmetric compartments [11]. The nuclei are transported on microtubule tracks by dynein motors to evenly spaced positions along the nascent germ tube [12]. After subsequent mitotic divisions, more septa are formed, further partitioning the hypha. Throughout extension and partitioning, mitosis continues in the tip cell, while mitosis is arrested in subapical compartments until the compartment branches [13,14]. To investigate the relative arrangement of daughter and non-daughter nuclei, we used an *A*. *nidulans* strain transformed with photoactivatable tandem dimer (td) Dendra2-histone H1 (Khang, in-prep). In six independent experiments, eight cells were observed in which a single nucleus was photoactivated before mitosis, and photoconverted histone H1 localization patterns were recorded after mitosis. In five germlings that contained two nuclei, either the distal or the basal nucleus was photoconverted. In this scenario, we expected that following mitosis the two nuclei derived from the activated nucleus would contain photoactivated histone H1. To our surprise, in four of five cells all nuclei contained activated histone H1 following one or two rounds of mitosis (Fig 1A and 1C, Table 1). Similarly photoactivation of a single nucleus in germlings containing four or eight nuclei also yielded a variety of patterns, including localization of activated histone H1 to two adjacent nuclei (presumably daughters), and to more than two adjacent nuclei (daughters and non-daughters) (Fig 1D–1F). The only pattern not observed was localization to nonadjacent nuclei (Table 1). The presence of photoactivated histone H1 in more than two nuclei after mitosis suggested that histone H1 diffuses between nuclei.

**Table 1:** Position of photoactivated Dendra2-histone H1 following mitosis.

Cell #	Pattern^a^	Pattern^b^
1	O**X**	**XXXX**
2	O**X**	**XXXX**
3	O**X**	**XXXX**
4	O**X**	**XXXXXXXX**
5	O**X**	OO**XX**
6*	OOOOOOO**X**	OOOOOOOOOOO**X**
7	O**X**OO	OO**XXX**OOO
8	OOOOO**X**OO	OOOOOOOOOO**XX**OOOO

“O” denotes non-activated histone H1. Asterisk (*) denotes cell where nuclei were observed in a non-mitotic cellular compartment and therefore did not undergo mitosis throughout the experiment. “X” denotes contains activated Dendra2-histone H1 (Bold).

^a^ Pattern of histone H1 in cells immediately following photoactivation.

^b^ Pattern of histone H1 in cells at the end of the experiment.

To determine whether histone H1 could also move between nuclei in the absence of mitosis, we photoactivated a single nucleus within a subapical, non-mitotic compartment which had already been partitioned by septa. We predicted that if mitosis is required for histone H1 movement we would see a single nucleus with photoactivated histone H1 even after several hours of incubation, while if it is dispensable, we would still see two or more nuclei carrying photoactivated histone H1. In one experiment, we photoactivated nuclei in two separate cells, one of which was photoactivated at the 2-nucleus stage in the basal-most portion of the germling, and the other cell activated at the 8-nucleus stage, within a basal compartment. Negligible localization of histone H1 to adjacent nuclei was observed within the hypha which had not undergone nuclear division, after approximately 3 hours of growth had elapsed (Fig 1G–1I and Table 1). The presence of Dendra2-histone H1 signal in the hypha starting from the two-nucleus stage was observed, however only in the putative ‘daughter nucleus’ after one nuclear division. Histone H1 protein signal was not visible in the non-daughter nuclei, as would be predicted in a hyphal compartment with no septa or obvious barriers for protein transport. These data from photoactivation experiments suggest that histone H1 proteins can localize to daughter and non-daughter nuclei within a hyphal compartment following mitosis. The localization process however appears to occur in a stochastic manner, producing heterogeneous patterns, consistent with diffusion of histone H1.

**Fig 2:**
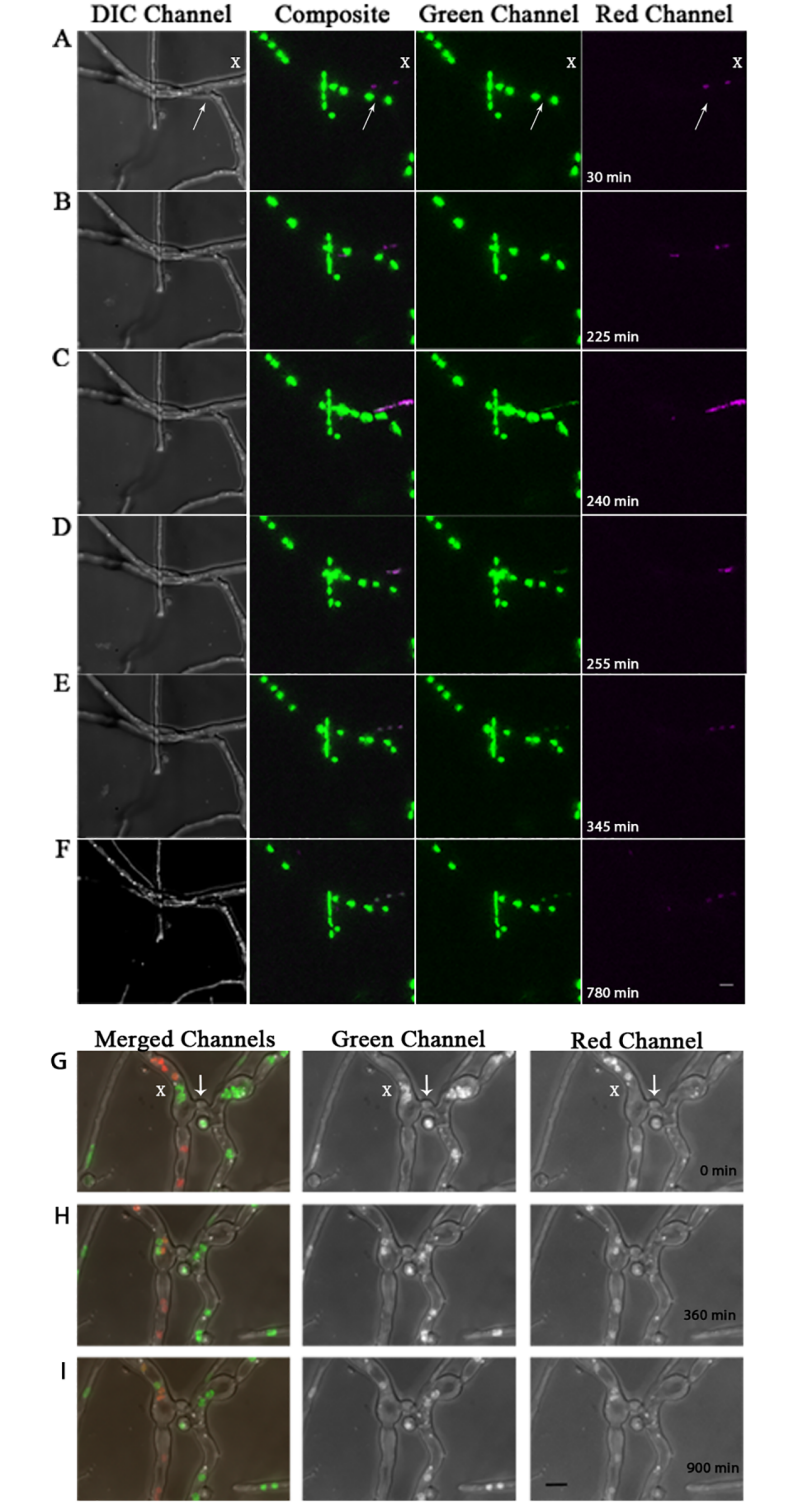
Histone H1 diffusion between genetically distinct nuclei can occur within cellular compartments of heterokaryotic mycelia. Still images of DIC, merged, green and red channels from supplemental video S2 Fig, showing histone H1 diffusion within vegetative mycelia of a heterokaryon fusion between histone H1-GFP and H1-RFP-containing strains. (A-F) Showing time points: [30min, 225min, 240min, 255min, 345min and 780min] from video S2 Fig respectively (15 min/frame). Still images of merged, green, and red channels from supplemental video S1 Fig show interaction between histone H1 within vegetative mycelia of H1-GFP and H1-RFP heterokaryon fusion. (G-I) Showing time points: [0min, 360min, and 900min] from video S1 Fig (15 min /frame). White arrows denote the junction of anastomosis. White X’s mark the cellular compartments where histone H1 diffusion between genetically distinct nuclei occurs. Scale bars = 5μm.

Experiments described above showing apparent diffusion of histone H1 between nuclei in *A*. *nidulans* made use of the constitutive expression of histone H1 gene from the filamentous fungus *Neurospora crassa* fused to the Dendra2, followed by photoactivation with a 405nm laser. To eliminate the possibility that the observed histone H1 localization to non-daughter nuclei resulted from use of a heterologous histone H1, the Dendra2 tag, or the 405nm laser, we exploited the ability of *A*. *nidulans* to form heterokaryons, a developmental state common in fungi in which genetically distinct nuclei co-exist in the same cellular compartment. Two strains were forced to produce a heterokaryon using complementary auxotrophic markers [15]. In addition to the auxotrophic markers, one strain carried the native *A*. *nidulans* histone H1 gene fused to RFP, and the other carried the native *A*. *nidulans* histone H1 gene fused to GFP. Time-lapse microscopy was conducted to visualize the nuclear interactions from each strain following anastomosis (hyphal fusion). If the apparently stochastic diffusion we observed was not a result of heterologous gene expression, the Dendra2 tag, or laser activation, we expected that GFP and RFP signals would colocalize in some heterokaryon nuclei, but not in all. In one time-lapse experiment we observed histone H1-GFP nuclei which had breached the junction of anastomosis and exchanged histone proteins as the nuclei moved past one another within the hyphal compartment (S1 Fig and Fig 2G–2I). In another time-lapse video, colocalization of histone H1 was observed to coincide with a mitotic event in an adjacent hyphal compartment, though nuclei did not make physical contact or traverse the junction of anastomosis into a ‘non-native’ hyphal compartment (S2 Fig and Fig 2A–2F). These time-lapse videos show a non-uniform/non-reciprocal exchange of histone H1-GFP and histone H1-RFP between nuclei of each strain.

**Fig 3:**
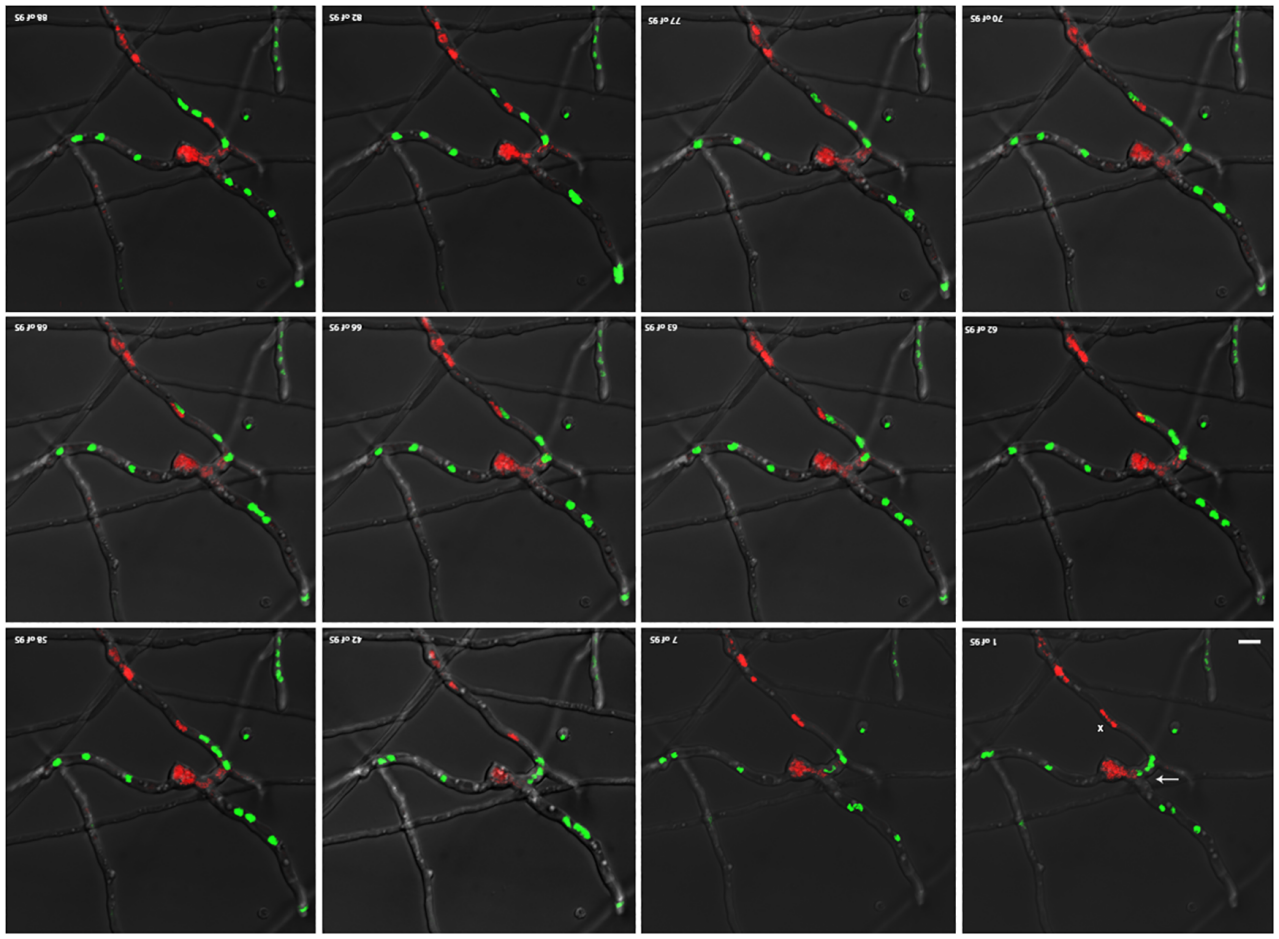
Genetically distinct nuclei can share cellular compartment of heterokaryotic mycelia without apparent histone H1 diffusion. Still images of merged red and green channels from supplemental video S3 Fig, showing histone H1 diffusion within vegetative mycelia of a heterokaryon fusion between histone H1-GFP and H1-RFP-containing strains. Images show frames [1/95, 7/95, 42/95, 58/95, 62/95, 63/95, 66/95, 68/95, 70/95, 77/95, 82/95, 88/95] from video S3 Fig respectively. (Continuous imaging between frames for the duration of approximately 95 minutes). White Arrow denotes the junction of anastomosis between homokaryotic mycelia. White X marks the cellular compartment where H1-GFP and H1-RFP-containing nuclei interact. White scale bar = 5μm.

In another time-lapse video, GFP-labeled nuclei from a heterokaryon between histone H1-GFP and histone H1-RFP strains were observed crossing the septal pore into the adjacent hyphal compartment directly following anastomosis (S3 Fig and Fig 3). In contrast to the previously described videos, S3 Fig and Fig 3 show genetically distinct nuclei sharing the same cytoplasm, shuffle past one another within the same hyphal compartment, without any apparent histone H1 diffusion.

One possibility for the lack of histone H1 diffusion for the duration of the time-lapse experiment in Fig 3 could be explained by the lack of mitosis. This process could be mitosis-dependent, requiring the partial disassembly of the nuclear envelope as a prelude for protein diffusion between nuclei. There were occasional cases however, where mitosis had occurred, but there was no observed histone H1 diffusion (Table 1, Cells 5, 6, and 8). In one of the experiments, the cell had undergone mitosis in a mitotically active compartment and the photoactivated nucleus remained in the non-mitotic compartment (Table 1, Cell 6). In the other experiments, there were mitotic events which took place in the compartment containing the photoactivated nucleus, but photoactivated histone H1 signal was only observed in a presumably daughter nucleus; although we cannot say definitively whether diffusion occurred between mother and daughter nuclei as well (Table 1, Cells 5 & 8). One possible explanation for this stochastic behavior of histone diffusion could be that there may be spatial or temporal constraints presented by the partial disassembly of the nuclear envelope in a semi-open, parasynchronous mitosis system of *A*. *nidulans* which dictates the movement of histone proteins between adjacent nuclei. Small differences in timing and nuclear spacing may have a significant impact on the level of histone H1 homogenization within a hyphal compartment; perhaps there is a certain proximity threshold for protein diffusion to take place between nuclei. It is also possible that diffusion had occurred, but was under the level of detection for these particular experiments.

The simplest explanation for the observed non-uniform histone H1 protein mixing is diffusion to adjacent nuclei across hyphal compartments following mitosis. Our use of the term ‘diffusion,’ refers to an observed movement of histone H1 proteins between adjacent nuclei that appears to be a passive process, affecting nuclei in close proximity. Confocal fluorescence micrographs show a gradient of colocalization of histone H1-RFP and histone H1-GFP signal between adjacent nuclei across hyphal compartments, suggesting a passive movement of proteins, although an active process of histone transport cannot be ruled out at this juncture (data not shown).

**Fig 4:**
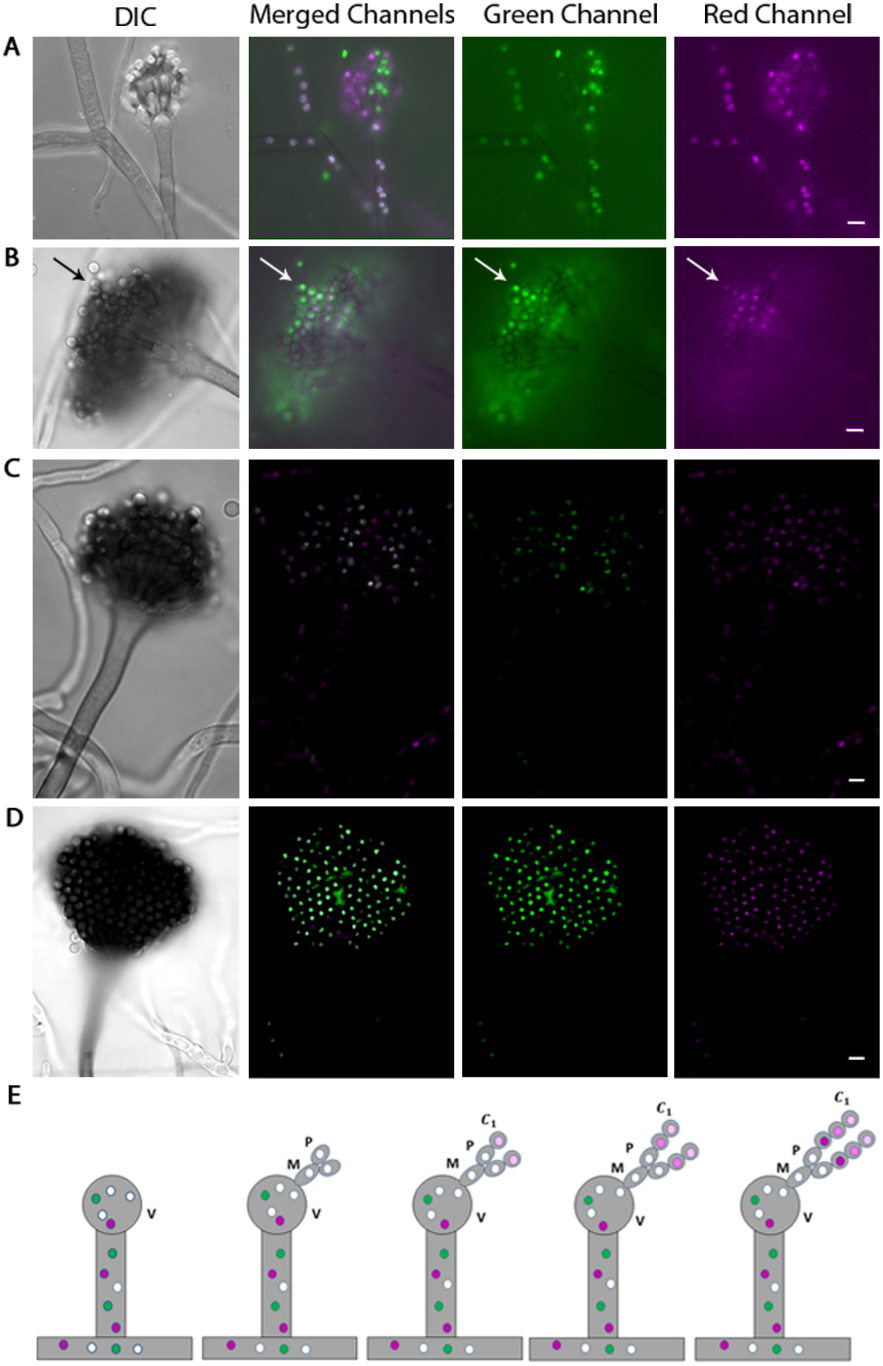
Conidiophores show a heterogeneous mixture of histone H1 protein as conidia develop. (A-D) Conidiophores derived from a heterokaryon fusion of H1GFP and H1RFP-containing strains imaged in DIC, merged, green, and red channels respectively. (D) Conidiophores derived from a heterokaryon fusion of H1GFP and H1RFP-containing strains which were selected for stable diploid colonies. (E) A postulated model of the histone H1 distribution within a single conidiophore derived from a heterokaryon fusion of histone H1-GFP and H1-RFP-containing strains (V = Vesicle, M = Metula, P = Phialide, C1 = First Conidium). Small colored circles in model represent nuclei; magenta and green circles represent nuclei with fluorescent signal from only histone H1-RFP or H1-GFP respectively. White circles represent nuclei showing a colocalization of fluorescent signal from histone H1-RFP and H1-GFP; all other circle colors represent a heterogeneous composition of fluorescent signals. The progression of darker shades of magenta nuclei within each newly-formed conidium in panel E represents the dilution of exogenous histone H1 protein (H1-GFP) relative to endogenous H1 protein (H1-RFP) as conidiogenesis occurs. All DIC micrographs were imaged in single focal plane. Panels A-B imaged in a single focal plane using a Zeiss Axioplan microscope. Panels C and D are a maximum projection of multiple Z-stack images, captured on a Zeiss LSM 510 VIS/META confocal microscope. White arrow indicates a chain of conidia showing heterogeneous mixture of histone H1-GFP and H1-RFP. White scale bars = 5μm.

An alternative explanation for the mixing of histone H1-GFP and histone H1-RFP in heterokaryon nuclei would be that diploidization and/or genetic recombination had occurred. To test these possibilities we used the process of asexual reproduction (conidiation) to resolve heterokaryon nuclei into individual spores (conidia) that could be tested. Uninucleate conidia are produced on specialized spore-bearing structures, called conidiophores, through a series of budding steps. Vesicle cells are formed from a septate vegetative hyphal compartment, and two additional layers subsequently emerge from the vesicle structure, the metulae and phialides. From the phialides, each nuclear division partitions a single nucleus into a budding conidium, forming a chain of uninucleate haploid conidia, each obtaining its nucleus from the original ‘stem cell-like’ phialide [16]. The spores closest in proximity to the phialide cells are more recently developed than those more distally oriented in the chain of conidia. Conidiophores from heterokaryon colonies were analyzed by fluorescence microscopy. If histone H1 protein diffuses between nuclei, we predicted that any yellow phialide nucleus would contain both the fluorescent signal encoded in its genome (endogenous), as well as diffused fluorescent protein from adjacent genetically distinct nuclei. Thus during conidiogenesis each conidium derived from that phialide would stochastically exhibit less ‘exogenous’ histone H1 protein as they are produced along a growing chain of conidia, as well as steady levels of ‘endogenous’ histone H1 protein (Fig 4E). In five independent experiments, observing approximately 50 conidiophores each, three permutations of histone H1 fluorescence signal heterogeneity were observed (Fig 4). One pattern observed was the colocalization of histones H1-RFP and H1-GFP, within individual nuclei, extending from the fused hyphal compartments up into the vesicle. The nuclei exhibit either histone H1-GFP or H1-RFP signal within the metulae, phialides, and chains of budding conidia, but colocalization is visible in the vesicle and vegetative hyphae (Fig 4A). This is likely an example of a conidiophore which has undergone a sufficient number of nuclear divisions to partition the exogenous histone H1 proteins. Another pattern observed was of histone H1 heterogeneity as colocalization of fluorescence signal among a chain of uninucleate spores (Fig 4B). The final pattern observed was of conidiophores with a heterogeneous mixture of both histone H1-GFP and histone H1-RFP throughout the entire spore-bearing structure (Fig 4C). A control for this experiment in which conidiophores were selected for stable diploid nuclei, showed a pattern where all nuclei within the spore-bearing structure exhibited both histone H1-GFP and histone H1-RFP (Fig 4D). These data together suggest that nuclei in each conidium can contain a heterogeneous mixture of both ‘endogenous’ and ‘exogenous’ histone proteins.

To determine whether the histone H1-GFP and histone H1-RFP heterokaryons showing a heterogeneous histone H1 pattern had undergone diploidization or genetic recombination, a genetic analysis of uninucleate conidia was conducted. The homokaryotic parent containing histone H1-RFP had the pyrG89 auxotrophic marker in its genotype, requiring the supplementation of Uridine/Uracil for growth on minimal media. The other homokaryotic parent containing histone H1-GFP had the pyroA4 and riboB2 auxotrophic markers in its genotype, requiring the supplementation of pyridoxine and riboflavin for growth on minimal media. If diploidization had taken place in the heterokaryon fusions prior to conidiation, uninucleate diploid spores derived from these colonies should grow as prototrophs. If genetic recombination had taken place in the heterokaryon fusions prior to conidiation, uninucleate haploid spores derived from these colonies should grow as prototrophs, or lose one or more auxotrophic markers in their genetic backgrounds. Positive growth of uninucleate haploid spores solely on media supplemented with the respective parental auxotrophic markers from each fungal strain suggests no diploidization or genetic recombination had taken place within the histone H1-RFP and histone H1-GFP heterokaryon fusions prior to conidiation (Table 2).

**Table 2:** Heterokaryotic nuclei do not show genetic recombination.

	Red Nuclei	Green Nuclei	MM + Uridine/Uracil	MM + Pyridoxine + Riboflavin	MM + Pyridoxine	MM + Riboflavin	MM
**Heterokaryon Parent # 1 (HA344)**	Yes	No	+	-	-	-	-
**Heterokaryon Parent #2 (LO1699)**	No	Yes	-	+	-	-	-
**Conidia derived from Heterokaryon between Parents #1 and #2**	Yes	No	98	0	0	0	0
	No	Yes	0	52	0	0	0

Marker scoring assay of uninucleate haploid conidia derived from a heterokaryon fusion of histone H1-RFP and histone H1-GFP strains HA344 (Heterokaryon parent # 1) and LO1699 (Heterokaryon Parent #2) respectively. Homokaryotic parental strain HA344 in heterokaryon fusion containing red nuclei has the genotype [*An-hH1-chRFP*::*pyroAAf;(pyroA4;argB2);pyrG89*;Δ*nkuA*::*argB*]. Homokaryotic parental strain LO1699 in heterokaryon fusion containing green nuclei has the genotype [*An-hH1-GFP*::*pyrGA*.*f*.; *(pyroA4;riboB2;argB2);pyrG89*; Δ*nkuA*::*argB*]. Heterokaryon fusions were generated between the two strains in fully supplemented complete media and all mycelial mats were transferred to selective minimal media. Conidia from heterokaryotic mycelia were harvested and replica-plated onto minimal media supplemented with all combinations of auxotrophic markers from each parental strain, and scored for (+/-) growth. Colonies were visually verified by fluorescence microscopy. Three independent experiments were conducted. N = 50.

Together the Dendra and heterokaryon data show that histone H1 stochastically migrates between non-daughter nuclei within hyphal compartments. In the case of the photoactivatable Dendra2 experiments, it is clear that the histone H1 protein itself diffuses among nuclei. The heterokaryon experiments are consistent with the idea of histone H1 protein diffusion between nuclei, though we cannot rule out the possibility that mRNA might also be exchanged in a stochastic manner. Although the details of histone diffusion are unclear, the experiments shown here suggest linker histone H1 undergoes dynamic internuclear movement, coinciding with mitosis. These experiments also suggest that care should be used when interpreting nuclear localization studies using histone H1 to track nuclear dynamics. It is tempting to speculate that fungi undergoing synchronized-open or semi-open mitosis might use histone diffusion to better homogenize adjacent nuclei within a single hyphal compartment, while fungi with an asynchronous, closed mitosis might use nuclear transport via cytoplasmic flow for the same end. The phenomenon of internuclear transfer of histone proteins could be a general mechanism in filamentous fungi to allow sharing of epigenetic information within fungal colonies. Future experiments should address a more thorough analysis of histone movement across a range of mitotic behaviors which could translate to other filamentous fungal systems.

## Materials and methods

### Dendra2 transformation

The plasmid pCK1285 contains a fusion of the histone H1 gene from *N*. *crassa* and the tandem dimeric Dendra2 (H1:tdDendra2) under control of the *Magnaporthe oryzae* RP27 constitutive promoter (Khang, in-prep). The riboB2 gene from *A*. *fumigatus* was cloned into pCK1285, and the resulting plasmid was named pAM001. Plasmid pAM001 was verified by *Eco*RI digestion and diagnostic PCR using KOD XTREME Hot Start DNA Polymerase (71975–3, EMD Millipore) according to manufacturer’s instructions. Plasmid pAM001 containing hH1:tdDendra2<riboB2> was transformed into an *Aspergillus nidulans* Δ*nkuA* fungal strain ATKO6 to minimize random integration into the host genome, by chemical transformation as previously described [17,18]. The positive transformants containing the riboB2 auxotrophic marker were selected for putative integration of the plasmid DNA into the fungal host genome. Approximately 100 transformants were 3-phase streaked to isolate single colony forming units, and analyzed under 100X oil immersion to verify Dendra2 fluorescence, using a Zeiss Axioplan microscope and a Zeiss Axiocam MRc charge-coupled device camera and software as previously described. Four positive transformants which showed H1:tdDendra2 fluorescence signal were chosen to go through two additional rounds of growth on selective media and visually screened for maintenance of fluorescence signal to ensure the plasmid was stably inserted into the genome. After selection, each positive transformant was checked at three developmental stages and verified to be morphologically indistinguishable from parental strain ATK06 and wild type A850. Positive transformant AAM010 was used in this study for photo-conversion experiments. All strains and plasmids used in this study are listed in Table 3.

**Table 3:** Strains used in this study.

**Strain Number**	**Genotype**	**Source**
FGSC A850	*biA1;argB*::*trpC_B;methG1;veA1;trpC801;argB2*	FGSC
HA344	*An-hH1-chRFP*::*pyroAAf*;Δ*nkuA*::*argB;pyroA4;argB2;pyrG89*	S. Osmani
LO1699	*An-hH1-GFP*::*pyrGA*.*f*.;Δ*nkuA*::*argB;pyrG89;argB2;pyroA4;riboB2*	B. Oakley
ATK06	*wA;pyrG89;argB2;pyroA4;lysB5;nicA2;riboB2*	This study
AAM010	*hH1*:*(td)Dendra2*::*riboB2;wA;pyrG89;argB2;pyroA4;lysB5;nicA2;riboB2*	This study
**Plasmid Number**	**Genotype**	**Source**
pCK1285	*Hpt*:*TrpC;EcoRI;RP27*:*hH1*:*(td)Dendra2*	This study
pAM001	*Hpt*:*TrpC;EcoRI;RP27*:*hH1*:*(td)Dendra2*::*riboB2*	This study

### Photo-conversion

Approximately 1X10^6^ spores were equally distributed in 50μl droplets on thinly-poured, fully-supplemented solid minimal media agar. Flame-sterilized coverslips were placed on top of the spore droplets. Petri plates were incubated at 30°C until desired developmental stage was reached. The area of agar directly underneath the coverslips was dissected using a sterile scalpel and mounted onto flame-sterilized microscope slides. Each slide was viewed under 100X oil immersion objective lens on a Zeiss LSM 510 VIS/META confocal microscope at room temperature, with an upright AXIO Imager M1 microscope stand and Diode (405 nm), Argon (458, 477, 488, 514 nm), HeNe1 (543), HeNe2 (594) and HeNe3 (633 nm) laser lines. Using bleach settings on LSM Imaging software, a ROI was designated to highlight a particular nucleus, and photo-conversion was conducted at: 100–150 iterations, 2.56μsec pixel dwell time, at 75–100% 405nm laser power. Z-stack images were captured before conversion, after conversion, and specific time-intervals between each image. Brightness increased for entire panel images for Dendra2 experiments to improve visualization wherever necessary. Maximum intensity projections of z-stack images and image settings compiled and adjusted respectively in Image J 1.48v (Java 1.6.0_20 (64-bit)) and Zeiss LSM Image Browser software (Version 4,2,0,121).

### Heterokaryons

Approximately 1X10^5^ spores of both strains LO1699 (H1-GFP) and HA344 (H1-RFP) were co-inoculated in Costar 24-well culture plates containing liquid complete media supplemented for all auxotrophic markers and incubated for 48–72 hours. Hyphal mats were transferred to minimal media solid agar plates without supplements, in order to facilitate heterokaryon formation by utilizing each strains complementary auxotrophic marker(s). The plates containing hyphal mats were incubated at 23°C for 2–3 days or until wispy putative heterokaryotic mycelia were visible on the periphery of the hyphal mat. Sections of agar were excised from the areas directly adjacent to the hyphal mats and mounted onto flame-sterilized glass microscope slides for observation. Selection for stable diploid mycelia was conducted by making 3 additional rounds of agar transfers followed by subsequent verification by fluorescence microscopy for colocalization of red and green fluorescence signal in all nuclei as well as growth on minimal media without supplements, verified by auxotrophic marker scoring assays. Strain HA344 (H1-RFP) was previously published [19]. Strain LO1699 contains *A*. *nidulans* histone H1 fused at its C terminus to a plant-adapted GFP [20] via a 5-GA linker constructed by fusion PCR as described in [21].

### Time-lapse microscopy

Strains LO1699 (H1-GFP) and HA344 (H1-RFP) were co-inoculated in Ibidi 8-microwell plates containing 300μL of liquid complete medium, supplemented with complementary auxotrophic markers, and incubated for approximately 16 hours at 30°C. The liquid complete media was drained from each micro well and washed 3X with ddH2O to remove residual media, and replaced with the same volume of liquid minimal media supplemented with only shared auxotrophic markers between the two strains. Wells were incubated for 8hrs at 23°C. Time-lapse images were taken every 15 minutes for approximately 15 hours, incubated at 30°C, under 100X oil immersion lens on a Deltavision Microscopy System I (pd125225) Olympus IX-71 inverted microscope. Filter sets used were POL, FITC, and TRITC for bright field, green fluorescence, and red fluorescence respectively. Videos were deconvolved and max projection images were produced using Deltavision Softworx 6.5.1 software. Videos were further processed using ImageJ 1.48v; Java 1.6.0_20 [64-bit] software.

### Marker scoring assay

Heterokaryon fusions of H1-GFP (LO1699) and H1-RFP (HA344) strains were cultured as previously described and allowed to conidiate. Conidia were harvested in ddH20, from excised sections of agar, and plated onto fully supplemented complete media. Two trials containing three biological replicates of 50 colony forming units each were replica-plated onto fully supplemented complete media and minimal media plates supplemented with all combinations of auxotrophic markers from each strain. Two independent trials containing 50 colony forming units each from selected stable diploids, were replica-plated on fully supplemented complete media agar plates and minimal media plates supplemented with all combinations of auxotrophic markers from each strain. Colonies were scored for growth, and verified for fluorescence signal under 100X oil immersion objective lens, using a Zeiss Axioplan microscope.

## Supporting information

S1 Fig Time-lapse video S1. (AVI)

S2 Fig Time-lapse video S2. (AVI)

S3 Fig Time-lapse video S3. (AVI)

S4 Fig Merged, green, and red channels of Fig 3, panels 1 & 12. (TIF)
