# Supplementary figures and images for: Internuclear diffusion of histone H1 within cellular compartments of Aspergillus nidulans
Source: PLoS One. 2018 Aug 16;13(8):e0201828. doi: 10.1371/journal.pone.0201828 (PMC6095493; doi:10.1371/journal.pone.0201828)

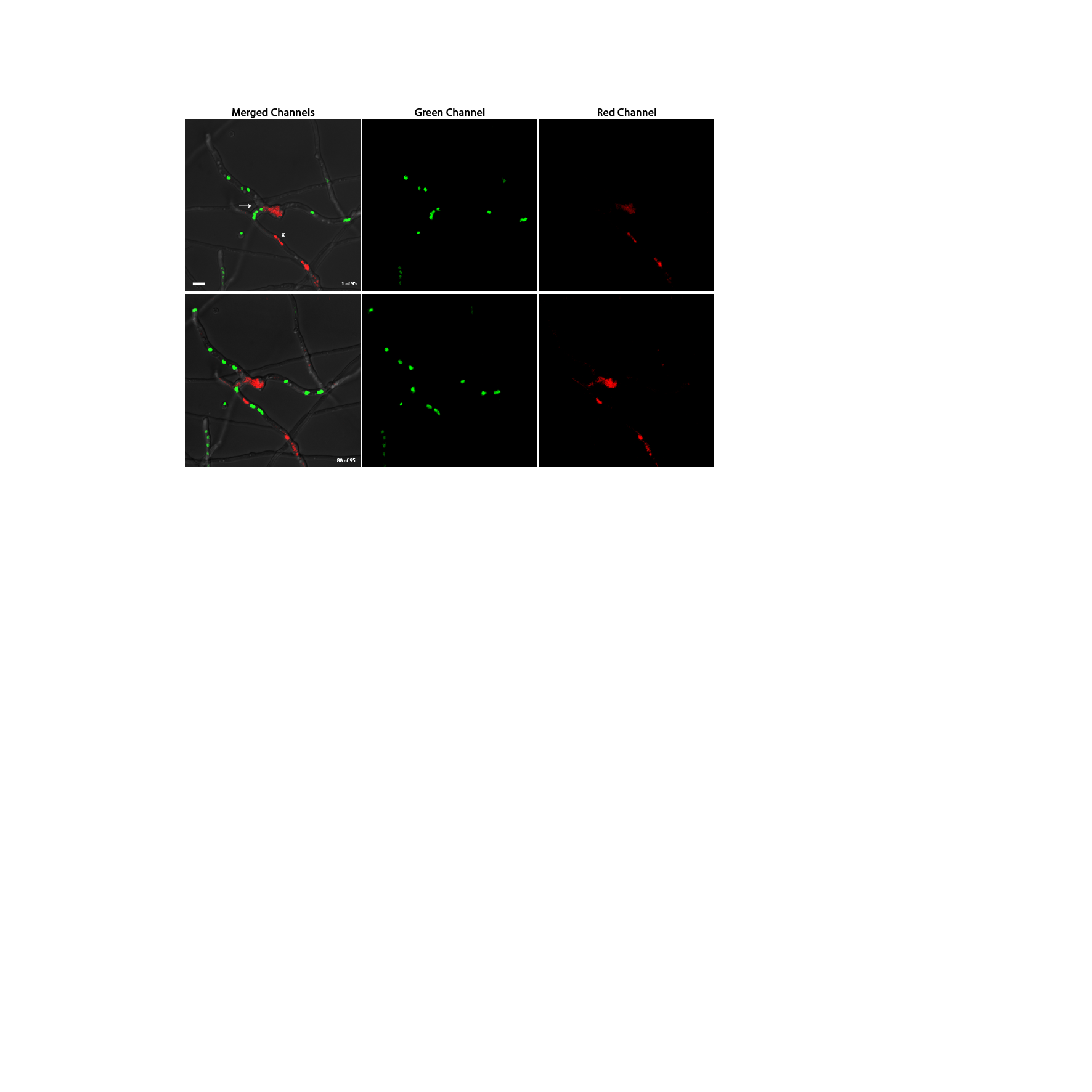

Supplement: S4 Fig — (TIF) [file pone.0201828.s004.tif]
